# Adaptive Clustering of Users in Power Domain NOMA

**DOI:** 10.3390/s23115314

**Published:** 2023-06-03

**Authors:** Yuri P. Santos, Luiz F. Q. Silveira

**Affiliations:** 1 Center for Research and Innovation in Information Technology, Federal University of Rio Grande do Norte, Natal 59077-080, RN, Brazil; yuripedro@dca.ufrn.br; 2 Department of Computer Engineering and Automation, Federal University of Rio Grande do Norte, Natal 59077-080, RN, Brazil

**Keywords:** non-orthogonal multiple access (NOMA), orthogonal multiple access (OMA), user’s clustering, DenStream, data rate optimization

## Abstract

By enabling multiple non-orthogonal transmissions, power domain non-orthogonal multiple access (PD-NOMA) potentially increases a system’s spectral efficiency. This technique can become an alternative for future generations of wireless communication networks. The efficiency of this method fundamentally depends on two previous processing steps: an appropriate grouping of users (transmission candidates) as a function of the channel gains and the choice of power levels that will be used to transmit each signal. Thus far, the solutions presented in the literature to address the problems of user clustering and power allocation do not consider the dynamics of communication systems, i.e., the temporal variation in the number of users and the channel conditions. In order to consider these dynamic characteristics in the clustering of users in NOMA systems, this work proposes a new clustering technique based on a modification of the DenStream evolutionary algorithm, chosen for its evolutionary capacity, noise robustness and online processing. We evaluated the performance of the proposed clustering technique considering, for simplicity, the use of an already widely known power allocation strategy called improved fractional strategy power allocation (IFSPA). The results show that the proposed clustering technique can follow the system dynamics, clustering all users and favoring the uniformity of the transmission rate between the clusters. Compared to orthogonal multiple access (OMA) systems, the proposed model’s gain was approximately 10%, obtained on a challenging communication scenario for NOMA systems since the channel model adopted does not favor a large difference in the channel gains between users.

## 1. Introduction

Since the first generation of mobile networks, multiple access techniques have been based on the same orthogonal multiple access paradigm. In this paradigm, each user accesses the system using an orthogonal communication approach, in either the code, time, frequency or space domain. Specifically, frequency division multiple access (FDMA) was used in the first generation (1G), while time division multiple access (TDMA) and code division multiple access (CDMA) were used in the second generation (2G).

CDMA was also present in the third generation (3G) alongside GSM systems—the latter based on a combination of FDMA and TDMA. Orthogonal frequency division multiple access (OFDMA) was present in the fourth (4G) and fifth generations (5G) [1,2] as well as space division multiple access (SDMA) [3]. Despite being used since the first generation of mobile networks, schemes based on orthogonal multiple access are limited to the amount of orthogonal resources.

New multiple access techniques have been studied in order to improve the spectral efficiency of wireless systems [4,5,6]; among them, non-orthogonal multiple access (NOMA) emerged as a promising strategy for new generations of cellular networks [7]. By enabling non-orthogonal multiple access, NOMA supports more users per orthogonal resources available in the system.

Thus, it significantly improves the spectral efficiency of mobile communication systems [8]. NOMA systems can be classified into two categories [9]: power domain NOMA (PD-NOMA), considered in this work, and code domain NOMA (CD-NOMA). Although both applications in communications networks are relatively new, many approaches have already been proposed [10,11,12,13].

Power domain NOMA uses two techniques already known in the literature, superposition coding (SC) and successive interference cancellation (SIC), to enable the transmission of more than one user simultaneously using the same time-frequency-code resource. However, the success of interference cancellation in the receivers depends directly on power allocation schemes and user clustering.

A more significant difference in channel gains between grouped users favors a higher global transmission rate of the system. Specifically, when allocating more power to those users with worse channel conditions, it is possible to increase the global transmission rate of the system. Thus, a careful adjustment in the users’ power allocation and the decision of how the users share the system resources (defined by the clusterization method) allows the base station to be able to control, with flexibility, the general data rate, the cell edge data rate and ensure user fairness [2].

In fact, for a NOMA system to achieve its best performance, it would be necessary to evaluate all possible combinations of users in clusters and their respective power levels, which would be infeasible in computational terms. In recent years [14,15,16,17,18], some works on NOMA have focused on sub-optimal solutions for both problems, seeking to improve the system’s spectral efficiency and reduce interference between users.

In order to mitigate the multiple access interference in downlink NOMA, an approach based on beamforming was employed in [19]. Each cluster can be dealt with independently with the inter-cluster interference suppressed by beamforming. Thus, the problem is reduced to finding the optimal beamforming vector to minimize the transmit power for a given quality of service (QoS) in terms of target signal-to-interference-plus-noise ratios (SINRs) or transmission rates. The paper focused on the beamforming method and used the well-known user-clustering method [20] based on paring users with the maximum channel gain difference.

Although providing a significant increase in spectral efficiency, NOMA systems also increase energy consumption. Therefore, in [21], a solution for user clustering and power allocation was studied to maximize the energy efficiency under maximal transmission power, minimum data transmission rate requirement and SIC requirement. The clustering method has two steps: initially, pairs of users with the most significant differences between channel gains are formed, and each group is associated with a cluster. By Lagrange multipliers, each power coefficient is determined.

The authors in [22] studied joint power control and user clustering for downlink NOMA to minimize the total power consumption by considering the transmission power and the decoding power of the users, which is ignored in many works. The problem is transformed into an equivalent problem with tractable constraints. Then, an optimization algorithm dealt with the reformulated problem using re-weighted $\iota_{1}$-norm minimization and majorization–minimization techniques.

The user clustering effects and power allocation in downlink and uplink NOMA systems were investigated by [23]. In this work, users are clustered in groups of two, four and six users, according to the difference in their channel’s gain, prioritizing users with the best channel conditions. In each group, the power coefficients of the users are optimized by Lagrange multipliers.

In [24], the authors investigated the impact of user clustering over the reachable diversity order in a downlink NOMA system. A joint user clustering and subcarrier allocation scheme was proposed with enhanced minimum diversity order. In the worst case, this method allows the user with the highest outage probability to reach the same order of diversity that would be obtained using an exhaustive search scheme.

Adopting a different approach from other works, the authors in [25] proposed a user clustering scheme in a hybrid downlink NOMA system based on a genetic algorithm. The objective was to maximize the overall system throughput with no limit of users in every cluster. In the simulations, it was observed in several scenarios that the proposed scheme approached the optimal solution for any number of users present in a cluster.

A joint optimization design of beamforming and power allocation in the downlink NOMA-based satellite-terrestrial integrated network (STIN) operating at mmWave band was proposed in [26]. A multiuser downlink framework was presented to integrate a system where satellite networks exploit multicast communication and coexist with a cellular network that implements NOMA. For this, a new user pairing scheme was presented based on channel gain and correlation. The new method obtained the optimal solution of beamforming weight vectors and power coefficients, thus, improving the computational efficiency.

In [27], a clustering model was proposed using the method of pairing two users or three users together and combining the signal-to-noise ratio (SNR) levels to decide on two or three pairings in order to ensure the quality of service (QoS) for users. The method is divided into two stages, the first clustering using the users’ spatial location information by the K-means algorithm. Then, maintenance of these clusters is conducted to separate those users that do not satisfy the clustering conditions and then attempt to rejoin or transmit them by OMA. The results showed that the proposed model guarantees the quality of service requirements.

An adaptive multi-user clustering algorithm was proposed in [28]. The proposed model uses a combination of the traditional model with an adaptation step. The traditional model consists of ordering users by channel gain value and grouping more users with higher gain values with those with lower gain values. While the adaptive step attempts to fix clusters that do not reach a minimum SINR difference constraint between users by splitting, regrouping or transmitting by OMA. As result, higher user data rates were achieved. It was also noted that an increase in the number of users in a cluster does not always lead to higher NOMA rates.

It is possible to observe some common aspects to these works. They do not consider the temporal variation of the users’ channel gain. In addition, the proposed clustering techniques are generally non-adaptive since the investigated systems’ intrinsic dynamics are commonly not considered. Even works that propose an adaptation of clusters attempt to correct groupings that were initially made erroneously, still require initial knowledge of how many clusters will be formed.

### 1.1. Proposed Solution and Contributions

Although several proposed approaches are in the literature, the NOMA approach was not included in the fifth generation of mobile systems (5G). In fact, the viability of NOMA systems depends on the efficiency of the techniques used for user clustering and power allocation. However, finding efficient clustering and power allocation approaches that suit different communication scenarios remains challenging. In this context, a fundamental attribute of the user-clustering method is that it can dynamically adapt the clusters to the dynamics of the communication systems, whether due to the entry of new users into the network or the variation of the communication channel over time. To the authors’ knowledge, this has not been considered in the literature.

Thus, in this work, we present an adaptive user clustering, which uses an evolutionary algorithm based on the DenStream algorithm in one of its stages. Evolutionary algorithms allow the creation of new clusters and their adaptation, i.e., it is adaptable to the number of users on the network and to the variation of the state of each user’s channel, which is the feature used to perform the clustering.

For the power allocation, the IFSPA algorithm is used. This algorithm is a dynamic method that is suitable for the mobile environment. The IFSPA has already proved to be the power allocation algorithm that best reconciles sum-rate capacity, fairness and bit error rate (BER) [7]. Next, the contributions of this work are detailed:

- We propose using an adaptive clustering method, which considers the time-varying behavior of a mobile network, to increase the overall data rate of a NOMA downlink.
- We use an algorithm based on the DenStream to develop an adaptive and evolutionary clustering strategy, which adjusts to temporal variations in the channel state of each network user and the arrival of new users to the network, considering a downlink NOMA.
- We propose using a global fading to model the channel of each user, which considers a non-line-of-sight large-scale fading, modeled by COST 231 Hata, and a small-scale fading, modeled by the complex symmetric Gaussian.
- We evaluate the performance of the proposed solution for clustering and power allocation in downlink NOMA systems through the overall throughput.

### 1.2. Paper Organization

This paper is organized as follows: Section 2 presents the fundamentals of downlink NOMA systems. Section 3 describes the models used to generate the fading channel gains. In Section 4, we describe the proposed clusterization algorithm. Section 5 details the proposed model, including the clustering and power allocation algorithms. Finally, the results of the experiments are discussed in Section 6, including an evaluation of the system performance. Our conclusions for the work are presented in Section 7.

## 2. Downlink NOMA Fundamentals

The main idea of NOMA is to transmit to a number of users greater than the number of orthogonal resources of time, frequency or code resources available in the system. Two approaches for implementing NOMA can be found in the literature: NOMA in the power domain and NOMA in the code domain. In this work, we only consider NOMA in the power domain. From this point forward, any mention of a NOMA system pertains to NOMA in the power domain unless expressly stated otherwise.

NOMA explores the differences in channel gains between users for the multiplexing signals in the power domain. This technique seeks to at least double the number of devices connected simultaneously and achieve superior user and system performance gains [29] compared to conventional multiple access techniques. Based on information theory, it is known that non-orthogonal multiplexing using superposition coding at the transmitter and SIC at the receiver outperforms traditional orthogonal multiplexing and is also ideal for reaching the capacity region of downlink transmission channels [30].

In the downlink scenario discussed in this work, the transmitter generates different signals for different users so that they are superimposed on each other after the encoding and modulation steps. By sharing time, frequency and code resources among users, it is necessary to use multi-user detection (MUD) algorithms, such as SIC, to detect the different signals at the receiver [2].

**Figure 1:**
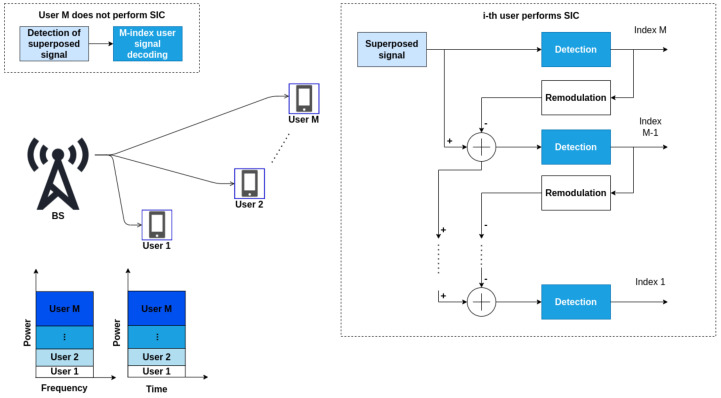
NOMA downlink example formed by one base station and M users.

To better understand how a NOMA system works, let us build a NOMA downlink scenario formed by a base station (BS) with a single antenna and *M* users with a single antenna each as illustrated in Figure 1.

The BS transmits the signal *x* simultaneously to different users. This signal is formed by the superposition of the signals $x_{i}$ intended for each user, weighted by a power normalization coefficient $p_{i}$, respectively, associated with the *i*-th user, according to:

(1) $$x=\sum_{i=1}^{M}\sqrt{p_{i}}x_{i},$$

where $\sum_{i=1}^{M}p_{i}=P$ with *P* as the total power of the transmitter. The following rule must be respected to define a power coefficient for a user: users with a weak channel coefficient must receive more power than those with a strong channel coefficient.

Therefore, the signal received by the *i*-th user can be defined by:

(2) $$y_{i}=h_{i}x+n_{i},i=1,2,\ldots ,M,$$

where $h_{i}$ denotes the channel gain between the BS and the *i*-th user, and $n_{i}$ represents the additive white Gaussian noise (AWGN).

After receiving $y_{i}$, the receiver uses the SIC to perform multi-user detection. The algorithm follows a detection order that is determined by the power coefficients assigned to each user, which is determined by the gain of the normalized channels of their respective devices $\left(|h_{1}{|}^{2}/N_{1}>|h_{2}{|}^{2}/N_{2}>\ldots >|h_{M}{|}^{2}/N_{M}\right)$, i.e., from the user with the weakest (high attenuation) to the strongest channel gain (low attenuation). It is important to emphasize that the user who receives the highest power can detect its signal and treat the other signals as noise. However, the other users will perform SIC until they can decode their respective signals.

Thus, it is assumed that any user can detect their information without significant interference caused by signals from other users. Users with better channel conditions require less power but can correctly detect their data with high probability. Therefore, assuming perfectly error-free decoding of the interfering signals, the achievable rate for the *i*-th user (i=1,2,…,M) can be written as [2]:

(3) $$R_{i}=wB\log\left(\begin{array}{c} 1+\frac{p_{i}{\left|h_{i}\right|}^{2}}{wN_{i}B+\left(\sum_{j=i}^{i-1}p_{j}\right){\left|h_{i}\right|}^{2}} \end{array}\right),i=1,2,\ldots ,M,$$

where *B* is the bandwidth of each channel in Hertz, *w* is the number of available resource blocks, and $N_{i}$ is the power spectral density of the noise.

## 3. Global Fading Coefficient

The proposed clustering method uses information from the users’ channel state information to define the formation and evolution of clusters. In this work, the path gain associated with each user channel is defined in terms of a relative global fading among network users, which considers the small-scale and large-scale fading as defined by:

(4) $$v=\sqrt{PL_{d}-PL_{d_{0}}}h,$$

where *h* is the small-scale fading, and $PL_{d}$ is the path loss to a distance *d* from the base station, defined by the COST 231 Hata model as follows:

(5) $$\begin{array}{c} PL_{d}=-46.3-33.9\log_{10}\left(f_{c}\right)+13.82\log_{10}\left(h_{t}\right)+\alpha \left(f_{c},h_{r}\right)\\ -44.9+6.55\log_{10}\left(h_{t}\right)\log_{10}\left(d\right), \end{array}$$

where *d* is the distance between the base station and the user’s equipment in kilometers, $f_{c}$ is the carrier frequency in MHz, and $h_{t}$ and $h_{r}$ are the base station antenna and mobile station antenna effective height in meters, respectively. $\alpha (f_{c},h_{r})$ is the mobile station antenna height correction factor for suburban or rural environments, defined by:

(6) $$\begin{array}{c} \alpha \left(f_{c},h_{r}\right)=\left[1.1\log_{10}\left(f_{c}\right)-0.7\right]h_{r}\\ -[1.56\log_{10}\left(f_{c}\right)-0.8]. \end{array}$$

The small-scale fading, *h*, is defined by a time-varying complex Gaussian process generated by the Monte Carlo method, which has Rayleigh distribution in magnitude and uniform distribution in phase and power spectrum density as defined by [31]:

(7) $$H\left(f\right)=\left\{\begin{array}{cc} \frac{1}{\sqrt{1-(\frac{f}{f_{D}}}{)}^{2}}, & if\;|f|<f_{D}\\ 0, & if\;|f|\geq f_{D} \end{array}\right.$$

where $f_{D}$ is the maximum Doppler shift.

The clustering algorithm proposed here considers the relative global fading among network users to define the clusters over time. Therefore, the path loss of each user in (4) is calculated relative to a common reference distance from the base station, $d_{0}$.

## 4. User Clustering Algorithm

Evolutionary algorithms are commonly used in data streaming in problems where the number of clusters is unknown, no data characteristics are known, and the number of clusters is a dynamic parameter. The issue of clustering users in NOMA networks has these characteristics, although most works address a fixed number of clusters. Therefore, in this work, we propose an algorithm based on the DenStream evolutionary algorithm, defined by [32], to solve the problem of clustering users in NOMA systems. The proposed algorithm can adapt to the arrival of new users, i.e., it creates new clusters without running the algorithm for each new user of the system.

The algorithm can be divided into two stages: an online stage, responsible for maintaining its clusters, and an offline stage, responsible for generating the final clusters and defining the power coefficient of each user in response to a BS demand.

**Figure 2:**
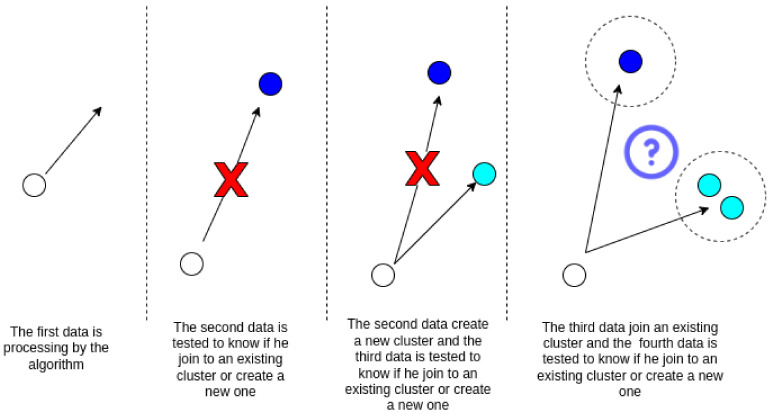
Proposal clustering algorithm. No cluster exists when starting the algorithm, so one is created for the user who arrives on the network. Then, new users arrive and are grouped according to their features.

Every time a user arrives, an attempt to merge with an existing cluster is made. If this is not possible, as it does not meet the requirements of the existing clusters, a new cluster is created for this user as illustrated in Figure 2.

**Figure 3:**
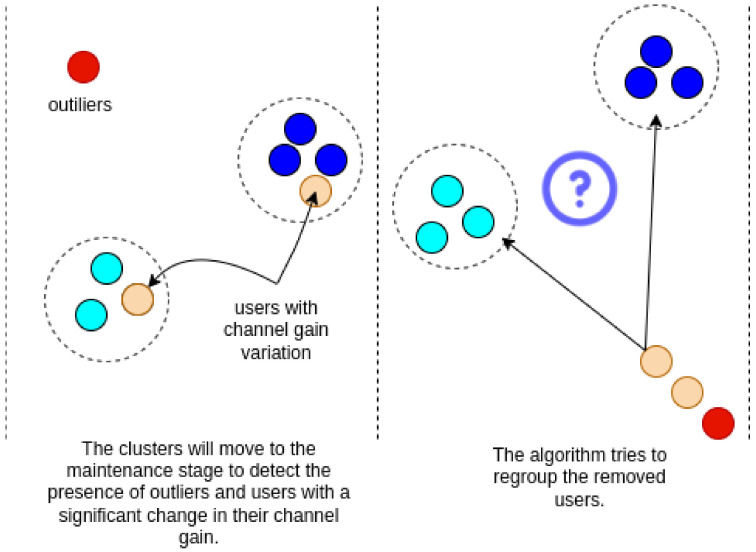
Cluster maintenance step. Each cluster is analyzed for outlier detection and channel gain variation in each user.

At each period t=T, where t=[0,∞) is the current instant of time, maintenance of the existing clusters is performed as illustrated in Figure 3. The proposed algorithm is sensitive to outliers and changes in the user’s channel gain. Unlike the original DenStream, the proposed method does not discard the user when they have a significant channel gain change; they are removed from the cluster and reinserted into the system’s input queue. The same happens with users that are detected as outliers to confirm that that the outlier was not mislabeled.

Different from the original DenStream, in which clusters are defined by weight (*W*), center (*C*) and radius (*Ray*). The proposed method replaces the center and radius with the channel gain list of each user (*Lg*) and the estimation of the gain of each user in time (*Lg(t)*). These clusters are classified as potential clusters (p-clusters) and outlier clusters (o-clusters); p-clusters can become final clusters, and o-clusters are treated as noise.

A new user *i* can join a p-cluster *j* if, with its inclusion, the standard deviation (σ) of the cluster is greater than η and less than φ, both given in percent, and the cluster satisfies the SIC constraint as defined by:

(8) $$\left(\begin{array}{c} p_{i}-\sum_{k=1}^{i-1}p_{k} \end{array}\right)\gamma_{i-1}\geq P_{tol},\forall_{i\in j},$$

where $p_{i}$ is the power of user *i*, and $P_{tol}$ is the minimum power difference required to distinguish between the signal to be decoded and the remaining non-decoded message signals. If more than one cluster satisfies the restrictions, priority will be given to the one with the least number of users.

If no p-cluster can cluster a new user, the system joins the new user to an o-cluster with the same restriction described above. If the user does not meet the conditions to join any clusters, the system creates a new o-cluster to cluster this user as seen in Algorithm 1. As it is an evolving data stream, the roles of clusters and outliers can change as new users arrive in the communication system because when new users are grouped in an o-cluster, its weight ($W_{j}$) increases as defined by:

(9) $$W_{j,new}=\sum_{m=1}^{M}w_{j,m}f\left(t\right)+w_{new},$$

where $f\left(t\right)=2^{-\lambda t}$ is called the forgetting function, for λ>0. This function controls the speed at which the o-clusters become irrelevant. The higher the value, the faster the weight of o-clusters decreases, decreasing the chances of making it a p-cluster. $w_{new}=1.0$ is the weight of the user *i* that will be added to the cluster *j*, and $w_{j,i}$ is the weight of the user *i* present in the cluster *j*.

If $W_{j}$ exceeds a threshold κ, the o-cluster *j* becomes a p-cluster with κ as the parameter that determines the outlier threshold. The smaller the κ, the more sensitive the cluster is to the arrival of new users; it only takes a few users for an o-cluster to become a p-cluster.
**Algorithm 1:** MergingAttempt to join user *i* to the nearest p-cluster $C_{p}$;
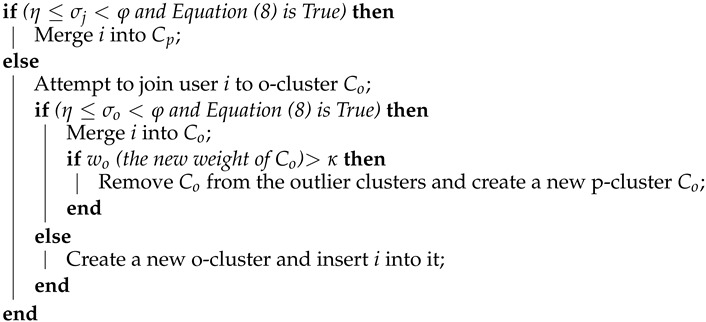


Every *T* seconds, each user in a cluster is analyzed for changes in their channel state. If the difference between the current channel gain of the *i*-th user, $Lg_{i}\left(t\right)$, and their old one, $Lg_{i}\left(t\right)\left(t-T\right)$, exceeds a threshold ζ, given in percentage, then the *i*-th user is removed from their cluster and added to the system’s input queue. At the same time, *T*, the elements in o-clusters $C_{o}$ are added to the system’s input queue as can be seen from Algorithm 2, where *T* can be defined by:

(10) $$T=\left\lceil \frac{1}{\lambda }\log\left(\begin{array}{c} \frac{\kappa }{\kappa -1} \end{array}\right)\right\rceil ,$$

and when the BS requires, in the offline step, the algorithm returns the final clusters formed by the cluster index of each group.
**Algorithm 2:** Modified DenStream*Calculate T by Equation (10);**Get the next user i at the current time t from the input data;*
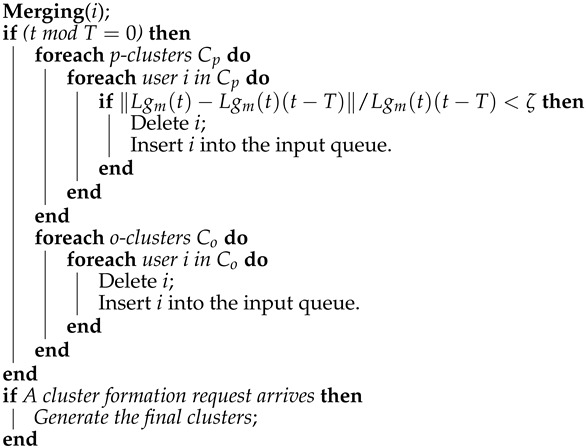


## 5. Proposed Model

**Figure 4:**
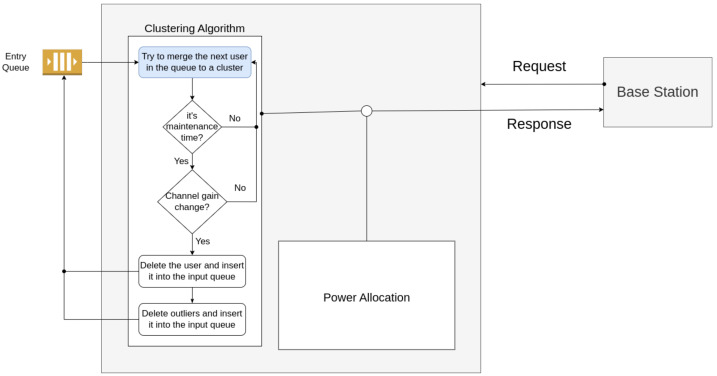
Flowchart of the proposed method.

This section presents the proposed model for user clustering and power allocation in a NOMA network, which is illustrated in Figure 4. At time t=0, each channel gain of each user equipment, Equation (4), is stored in the entry queue; after this, the proposed Algorithm 2 is executed online to obtain the firsts clusters formation.

Each user in the entry queue attempts to merge in an existing cluster using Algorithm 1. Every *T* period, each user in a p-cluster is verified. If any channel gain value of each user has a variation ζ in this period, this user is removed from the cluster and reinserted in the entry queue. If a user is identified as an outlier in period *T*, this user is removed from the o-cluster and added to the input queue.

At each transmission request of BS, the users in each cluster are associated with the power coefficients determined by the dynamic power allocation of the IFSPA technique [7]. Thus, the power coefficient of a user *i* in a cluster *j*, within a total of *M* users, is defined by:

(11) $$p_{j,i}=\frac{{\left(\frac{∥h_{j,M}∥}{∥h_{j,i}∥}\right)}^{\alpha_{j}}}{\sum_{n=1}^{M}{\left(\frac{∥h_{j,M}∥}{∥h_{j,n}∥}\right)}^{\alpha_{j}}},$$

where $∥h_{j,M}∥$ denotes the highest channel gain of the cluster *j*, $∥h_{j,i}∥$ denotes the gain of the user who is calculating the power coefficient, $\sum_{n=1}^{M}\frac{1}{∥h_{j,n}∥}$ is the sum of the gain of all users in the cluster *j*, and $\alpha_{j}$ is the power control parameter of the cluster *j*. The greater the value of α, the higher the power coefficient of the user with the weakest channel gain. After this, the BS is ready to transmit to its users on a NOMA network.

In this model, cluster formation adapts to the new users’ features; retraining is unnecessary. The execution of the evolutionary clustering strategy takes place in the BS. From the estimates of the user’s channel state that arrives at the BS, the clusters are updated or formed, the power coefficients of the users for downlink transmission are calculated, and transmissions begin. Next, experiments to evaluate the proposed system are described, and the global transmission transfer rate is investigated for this system.

## 6. Results and Discussions

**Figure 5:**
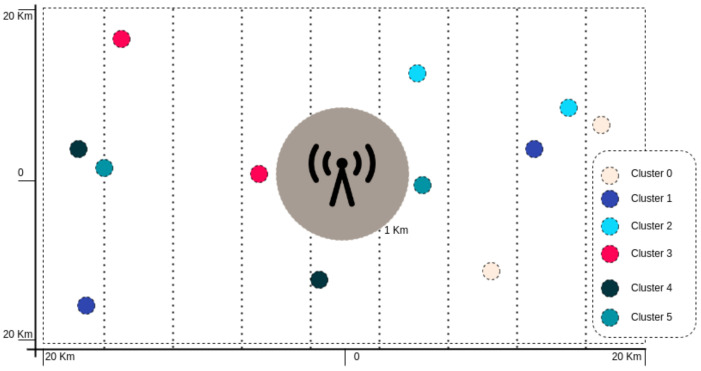
Distribution of users in space.

In the experiments, users are randomly distributed in a rectangular region around the BS. Figure 5 illustrates the user clustering based on their channel gains using the proposed clustering method.

The simulation parameters are shown in Table 1. The same values are typically found in the literature [23,25,33].

Users are considered to be randomly distributed in a region with distances from the BS ranging from 1 to 20 km, following typical average values. We also considered perfect knowledge of the channel state information in the experiments, using gain values defined by Equation (4) in 10,000-time stamps. The parameters used by the equation are presented in Table 2.

Table 3 presents the values adopted for the parameters of the proposed clustering algorithm and power allocation. These parameters were adjusted through several tests to maximize the performance of the proposed method.

### 6.1. The Experiment

**Figure 6:**
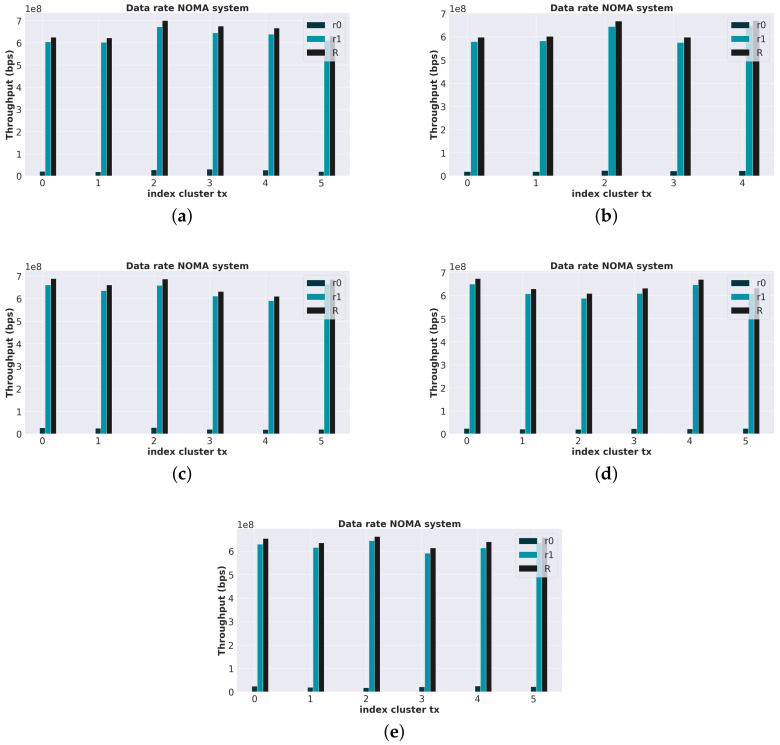
Time behavior of the throughput of a downlink NOMA system after the channel gain variation. (**a**) Throughput after new users enter the cell. (**b**) Throughput after 125 s of users in the cell. (**c**) Throughput after 241 s of users in the cell. (**d**) Throughput after 354 s of users in the cell. (**e**) Throughput after 439 s of users in the cell.

Initially, a network cell contains ten users served by a BS. Subsequently, new users join the cell, one at a time, totaling 12 users, a typical amount found in similar experiments in the literature and 3GPP [34]. In a period of *T* seconds, the cell’s input queue is checked to cluster new users and update who has experienced a significant change in channel gain. In this same period, a request for the final clusters is performed, the power coefficients are defined, and the throughput of each cluster is calculated as shown in Figure 6.

From Figure 6a–e, an improvement in the spectral efficiency of the system can be observed since the system maintained a high global data rate even with the entry of new users into the cell and with the variation of the users’ gain. It is also possible to notice, in Figure 6a–c, that the algorithm can create and delete clusters over time according to the evolution of the input data—that is, the channel gain values of the users. This is due to the cluster maintenance step present in the proposed algorithm, which is not possible in the methods found in the literature, as a fixed number of clusters is necessarily predefined.

A particular case occurs in Figure 6b. It is possible to notice that only ten of the twelve users are grouped. This happened because the two users did not satisfy the SIC constraint (Equation (8)) when being added to existing clusters, and a new one was not formed due to the similarity of their channel gains at that instant of time. However, note that, in Figure 6c, users who were outside groups were grouped in the following period due to evolving channel gain values.

From Table 4 and Table 5, we can analyze the average data rate over time by the number of users in the cluster and, thus, have a better idea of the impact of the channel gain changes over time. In Table 4, the users have channel gains that vary slowly with time, and this behavior allows the formation of clusters with three users, which does not occur in Table 5 when the variation is faster due to the increase in the maximum Doppler frequency shift.

It is also possible to notice that the proposed algorithm favors average data rate maximization when the number of users per cluster increases.

### 6.2. Comparison with OMA Systems

**Figure 7:**
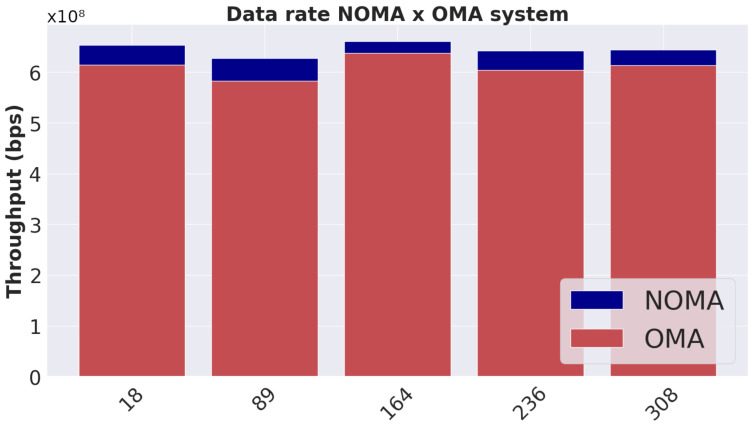
Behavior over time of the proposed model and OMA system.

Figure 7 shows the average sum data rate of NOMA and OMA systems for two user clusters. It is possible to note that there are advantages of a NOMA system with the proposed model as there is a consistency in the increase in the sum data rate over time when compared to OMA systems.

The slight variations over time that occur in Figure 7 are due to changes in the channel gain of each user. When users have more distant values of channel gains, the more significant the difference in the sum data rate is between a NOMA and OMA system and vice versa. The performance gains obtained in relation to the OMA in the analyzed time instants were equal to 6.3%, 8.5%, 4.6%, 5.9% and 5.0%. These values are characteristic of the channel model used, as this model does not favor a large difference in channel gain between users.

### 6.3. Comparison with Conventional Approach

The conventional method [35] for clustering users in a NOMA network consists of sorting users based on their channel gain values and grouping them in pairs following the near-far policy, i.e., grouping the user with the highest channel gain value with the one with the lowest channel gain value and, subsequently, performing the same procedure repeatedly for the other ordered users, until all users are grouped. This technique has some disadvantages, such as the re-run of the sorting and clustering algorithm for every sample that arrives and the prior need to define a fixed number of clusters in the system, apart from leading to a limited number of two users per cluster.

Such disadvantages of the conventional method can be circumvented with the proposed method in this article. The clustering algorithm works online, always adapting to new samples and is able to form a cluster of two or three users without the need for information on how many clusters should be formed. In addition, the proposed method is able to adapt the clusters as a function of the variation of the channel of each user in time without the need to re-run the algorithm.

Furthermore, as the proposed method is based on an algorithm used in data streams, it leads to the best configuration for that user that has recently arrived in the network, analyzing the scenario at that instant. For example, the first user that arrives at the network cell waits for the arrival of the next user to perform NOMA, or its signal is transmitted by OMA. After the arrival of a second user, the best possible configuration is to group the two users in the network cell if the SIC detector and outlier restrictions are satisfied. When a third user arrives in the network cell, there are two options, either a cluster of three users is formed, or the signal of this third user is transmitted by OMA until a fourth user arrives that can be grouped with this user. As the proposed method groups according to the state that the network is in after the arrival of a user, the conventional method and the proposed method generate different clusters of users.

However, compared to OMA, the proposed method needs to track the channel state information of each system’s user over time and has greater computational complexity.

### 6.4. Complexity Analysis

The most complex part of the proposed solution is the search for users in each cluster during the maintenance step, which has the worst-case complexity equal to O(n), where n is the number of clusters formed. As seen in the graphs above, due to the restriction of the SIC, it is favorable that there are more clusters of only two users so that the standard deviation calculation does not negatively affect the complexity of the system.

## 7. Conclusions

We presented a new method of clustering users based on evolutionary algorithms. The method was evaluated in a NOMA downlink scenario with dynamic power allocation. The evolutionary algorithm DenStream, which served as the basis for the proposed model, was chosen due to its ability to form clusters that adapt to new samples—that is, to new users who join a cell.

A relatively uniform overall data rate was observed in the results, even considering a temporal variation in the channel gain of all users and the ingress of new users into the cell, thereby, reinforcing the conclusion that the model is robust to these variations. Compared to OMA, the proposed system maintained the expected performance gain for NOMA, making better use of the dynamic power allocation method as it follows the channel state variations. The solution presents a competitive complexity concerning other solutions found in the literature, while it stands out for its online operation.

As the proposed method considered the state of the user’s channel to be clustered, a new user may not be grouped as soon as they arrive at the network cell if the channel state does not meet the system restrictions at that time. However, as the system tracks variations in each user’s communication channel, that user can still be grouped at a later time. The signal can still be transmitted by OMA. The system restrictions are adjusted by an initial parameterization that depends on the modeled communication channel.

The computational complexity of the proposed system increased linearly with the increase in the number of users; even so, it would be possible to use parallel processing to reduce the processing time of the method in networks with many users.

In future work, we intend to analyze the effects of correlation between channels over the proposed clustering method, which was not addressed in this work. Furthermore, we intend to optimize the proposed system considering a new dynamic power allocation method, and a study of algorithms based on game theory will be performed.

## Figures and Tables

**Table 1:** Simulation parameters for downlink NOMA.

Parameters	Values
Number of antennas in the BS	1
Number of antennas in the EU	1
Number of available resource blocks (w)	100
Resource block bandwidth (B)	180 kHz
Downlink transmission power budget ($P_{T}$)	46 dBm
Detection threshold at SIC receiver ($P_{tol}$)	10 dBm

**Table 2:** Parameters used to generate channel gain coefficients.

Parameters	Values
Carrier frequency ($f_{c}$)	2.0 GHz
Doppler frequency ($f_{d}$)	80 Hz
Base station antenna effective height ($h_{t}$)	30 m
Mobile station antenna effective height ($h_{r}$)	1.5 m

**Table 3:** Parameters of the proposed algorithm.

Parameters	Values
Forgetting factor (λ)	0.5
Outlier threshold (κ)	1.2
Sensitivity factor (ζ)	5%
Power control parameter (α)	0.2
Lower bound for grouping (η)	30%
Upper bound for grouping (φ)	$10^{3}\%$

**Table 4:** Average data rate and cluster formation in time with a 10 Hz doppler frequency.

Time	2-UEs	3-UEs
18.0	$6.48\times 10^{8}$ Bits/s	$6.69\times 10^{8}$ Bits/s
27.0	$6.52\times 10^{8}$ Bits/s	-
37.0	$6.43\times 10^{8}$ Bits/s	-
48.0	$6.50\times 10^{8}$ Bits/s	$6.72\times 10^{8}$ Bits/s
59.0	$6.27\times 10^{8}$ Bits/s	$7.00\times 10^{8}$ Bits/s
103.0	$6.47\times 10^{8}$ Bits/s	-
115.0	$6.39\times 10^{8}$ Bits/s	-
208.0	$6.44\times 10^{8}$ Bits/s	-
220.0	$6.50\times 10^{8}$ Bits/s	-
309.0	$6.51\times 10^{8}$ Bits/s	-
321.0	$6.37\times 10^{8}$ Bits/s	-
404.0	$6.35\times 10^{8}$ Bits/s	-
412.0	$6.35\times 10^{8}$ Bits/s	-
493.0	$6.35\times 10^{8}$ Bits/s	-

**Table 5:** Average data rate and cluster formation in time with an 80 Hz doppler frequency.

Time	2-UEs	3-UEs
18.0	$6.53\times 10^{8}$ Bits/s	-
27.0	$6.52\times 10^{8}$ Bits/s	-
37.0	$6.63\times 10^{8}$ Bits/s	-
48.0	$6.72\times 10^{8}$ Bits/s	-
59.0	$6.62\times 10^{8}$ Bits/s	-
103.0	$6.38\times 10^{8}$ Bits/s	-
115.0	$6.23\times 10^{8}$ Bits/s	-
208.0	$6.49\times 10^{8}$ Bits/s	-
220.0	$6.53\times 10^{8}$ Bits/s	-
309.0	$6.31\times 10^{8}$ Bits/s	-
321.0	$6.30\times 10^{8}$ Bits/s	-
404.0	$6.38\times 10^{8}$ Bits/s	-
412.0	$6.33\times 10^{8}$ Bits/s	-
493.0	$6.41\times 10^{8}$ Bits/s	-

## Data Availability

Not applicable.
